# Spin supersolid phase in coupled alternating spin chains

**DOI:** 10.1038/s41598-018-26169-y

**Published:** 2018-05-21

**Authors:** F. Heydarinasab, J. Abouie

**Affiliations:** 1 Department of Physics, Institute for Advanced Studies in Basic Sciences (IASBS), Zanjan, 45137-66731 Iran; 2 Department of Physics, Faculty of Science, University of Sistan and Baluchestan, Zahedan, Iran

## Abstract

We study the ground state phase diagram of a two dimensional mixed-spin system of coupled alternating spin-1 and 1/2 chains with a stripe supersolid phase. Utilizing different analytical and numerical approaches such as mean field approximation, cluster mean field theory and linear spin wave theory, we demonstrate that our system displays a rich ground state phase diagram including novel stripe supersolid, solids with different fillings and super-counterfluid phases, in addition to a stripe solid with half filling, superfluid and Mott insulating phases. In order to find a minimal mixed-spin model for stripe supersolidity, in the second part of the paper we consider two kinds of mixed-spin system of coupled alternating spin-1 and 1/2 chains with (i) anisotropic nearest neighbor interactions, (ii) anisotropic hoppings and study their ground state phase diagrams. We demonstrate that, for the systems with uniform hoppings, the repulsive intra-chains interactions are necessary for stripe supersolidity. In this case the minimal two dimensional mixed-spin model is a system of spin-1 and spin-1/2 XXZ chains, interacting via Ising Hamiltonian. In the case of anisotropic hoppings, a system of coupled Ising chains is the minimal model.

## Introduction

Supersolids are characterized by the coexistence of diagonal solid and off-diagonal superfluid long-range orders^1–4^. Combination of these two apparently antithetical properties has attracted the attentions of both experimentalists and theorists, and searching for this exotic phenomenon has become one of the main subjects of condensed matter and cold atoms physics^5–8^. Since the pioneering work of Jaksch *et al*., in describing the dynamics of an ultracold dilute gas of bosonic atoms in optical lattices with a Bose-Hubbard model^9^, lots of efforts have been devoted to search for supersolid phases in Bose-Hubbard models, experimentally and theoretically on one dimensional (1D) chains^10–13^, two dimensional (2D)^13–36^ lattice structures, bilayer systems of dipolar lattice bosons^37,38^ and three dimensional (3D) cubic lattices^17,39–41^.

Possible representations of bosonic operators with spin operators^42^, and bosonic statistics of magnetic excitations^43,44^ make spin systems another appropriate ground for searching various supersolid phases. It has been shown that 1D spin-1 chains^45–47^, 2D frustrated spin-1/2^48–59^ and spin-1^60–63^ models in an external magnetic field and 3D spin models^64,65^ possess different kinds of stripe, checkerboard and star supersolid phases. In these systems finite transverse magnetization implies the off-diagonal long-range superfluid order, the diagonal long-range solid orders are specified by longitudinal staggered magnetization, and the states with magnetization plateaux indicate the Mott insulating phases^43^.

In spite of many studies on uniform spin systems, the supersolidity of mixed-spin systems has not been addressed so far. Mixed-spin systems, or quantum ferrimagnets which are composed of different spins, mostly of two kinds, are a special class of spin models where their universality class is completely different from uniform spin models^66–69^. Ferrimagnets, which occur rather frequently in nature, are somehow between the antiferromagnets and the ferromagnets. Their lowest energy band is gapless which shows a ferromagnetic behavior while there is a finite gap to the next band above it which has the antiferromagnetic properties. It is the acoustical and optical nature of excitations which is the result of two different types of spin in each unit cell.

**Figure 1:**
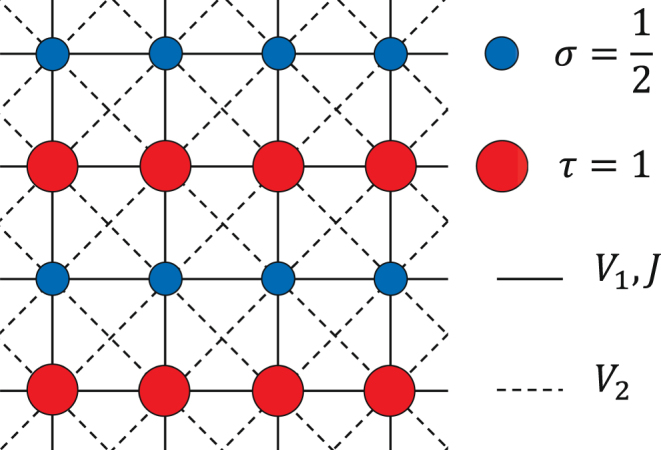
The schematic illustration of the coupled alternating spin-1 and 1/2 chains. The small (large) circles show spin $\sigma =\frac{1}{2}$ (*τ* = 1). The NN and NNN interactions are depicted by the solid and dashed lines, respectively.

Recently, we have studied a 2D frustrated ferrimagnetic spin model, originating from an inhomogeneous 2D bosonic system, composed of two kinds of hard-core (*a*) and semi-hard-core (*b*) bosons, respectively with the nilpotency conditions: ${({a}_{i}^{\dagger })}^{2}=0$ and ${({b}_{i}^{\dagger })}^{3}=0$, and shown that the model on a square lattice with nearest-neighbor (NN) and next-nearest-neighbor (NNN) interactions displays the checkerboard supersolid phase^70^ which is not observed in the 2D uniform spin-1/2 system on square lattices with short-range interactions^22,71,72^. Actually the interactions between spins with different sizes decrease the quantum fluctuations and cause the stabilization of the checkerboard supersolid order. Aside from the mentioned theoretical study, the spin supersolid phase has also been recently realized in the MnCr_2_S_4_ mixed-spin compound with spinel structure^73^. In this paper, we introduce a different system of coupled alternating spin *τ* = 1 and $\sigma =\frac{1}{2}$ chains (CAS) (see Fig. 1 and Eq. (1)) and show that our CAS system possesses a stripe supersolid (STS) phase, characterizing by the coexistence of stripe solid (ST) and superfluid (SF) orders. We investigate the ground state phase diagram of the CAS model using different analytical and numerical approaches such as mean field (MF) approximation, cluster mean field theory (CMFT) and linear spin wave theory (LSWT). Competition between NN and NNN interactions causes the system to undergo various first- and second-order phase transitions, and different solids, Mott insulators (MI), SF and super-counterfluid (SCF) to appear in the ground state phase diagram of the model, in addition to the STS. By studying the behavior of spin wave excitations, we investigate the stability of MF orders and demonstrate that, except at the superfluid-supersolid transition lines, overall quantum fluctuations are small in our CAS system and the MF predictions concerning the stability of phases are reliable.

In the second part of this paper, we look for a *minimal* mixed-spin CAS system, possessing an stable supersolid phase. In this respect, we consider two kinds of anisotropic CAS model: (*i*) a CAS system with anisotropic NN interactions where the intra-chains and inter-chains NN interactions are not the same, and (*ii*) a CAS system with anisotropic hopping energies in which the intra-chains and inter-chains hoppings are different. By obtaining the CMFT ground state phase diagrams of these systems, we demonstrate that the appearance of the STS order strongly depends on the amounts of intra-chains NN interaction. By studying the behavior of spin wave excitations, and also the behavior of diagonal and off-diagonal order parameters by CMFT with larger cluster sizes, we investigate amount of quantum fluctuations and consequently the stability of the STS phase in all anisotropic CAS systems. Our results indicate that the repulsive intra-chains NN interactions are necessary for the emergence of the STS phase. Finally, based on our achievements, we present a minimal mixed-spin CAS model with stable supersolid phase in the ground state phase diagram.

Our CAS model could be related to the mixed-valance iron polymer^74^ in the spin system and ladder-like optical lattices^75^ in the bosonic systems. Also our model could be realized in coupled one dimensional optical lattices^76^ by alternatively changing the optical depth.

This paper is organized as follows. In Sec. 2 we introduce our CAS system and present the bosonic counterpart of this model. In Sec. 3 we obtain the ground state phase diagram of the CAS model using MF approximation, CMFT and LSWT. The linear spin wave dispersions and number of excitation modes, are also presented in this section. The anisotropic CAS models with anisotropic NN interactions, and anisotropic hoppings are investigated in Sec. 4. In Sec. 5 we bring our methods and explain MF, CMFT and LSWT in detail. Finally, we will summarize our results and give the concluding remarks in Sec. 6.

## Our Model

### Coupled alternating spin 1 and $\frac{{\bf{1}}}{{\bf{2}}}$ chains

Let us consider a 2D system of coupled alternating spin *τ* = 1 and $\sigma =\frac{1}{2}$ chains (CAS), describing by the following Hamiltonian:

1 $$H={H}_{\sigma }+{H}_{\tau }+{H}_{\sigma \tau },$$

with

2 $$\begin{array}{rcl}{H}_{\sigma } & = & \sum _{\langle i,j\rangle ,\alpha }{{\mathscr{J}}}^{\alpha }{\sigma }_{i}^{\alpha }{\sigma }_{j}^{\alpha }-h\sum _{i}{\sigma }_{i}^{z},\\ {H}_{\tau } & = & \sum _{\langle i,j\rangle ,\alpha }{{\mathscr{J}}}^{\alpha }{\tau }_{i}^{\alpha }{\tau }_{j}^{\alpha }-h\sum _{i}{\tau }_{i}^{z},\\ {H}_{\sigma \tau } & = & \sum _{\langle i,j\rangle ,\alpha }{{\mathscr{J}}}^{\alpha }{\sigma }_{i}^{\alpha }{\tau }_{j}^{\alpha }+{V}_{2}\sum _{\langle \langle i,j\rangle \rangle }{\sigma }_{i}^{z}{\tau }_{j}^{z},\end{array}$$

where *α* = *x*, *y* and *z*. The Hamiltonians *H*_*σ*_, *H*_*τ*_, and *H*_*στ*_ include the intra-chains and inter-chains interactions, respectively. The parameters ${{\mathscr{J}}}^{x,y}$(=−2*J*) and ${{\mathscr{J}}}^{z}$(=*V*_1_) are the NN interactions, *V*_2_ denotes the inter-chains NNN interactions, and *h* is a magnetic field along *z* direction. The magnetic field *h* is proportional to applied magnetic fields as *h* = *g*_*σ*_*μ*_B_*B*_*σ*_ = *g*_*τ*_*μ*_B_*B*_*τ*_, where *μ*_B_ is the Bohr magneton, *g*_*σ*_ and *g*_*τ*_ are the g-factors, respectively, for spins-1/2 and spins-1, and *B*_*σ*_ and *B*_*τ*_ are the external magnetic fields applied to the subsystems with spins 1/2 and 1, respectively. Throughout this paper we consider *g*_*σ*_*B*_*σ*_ = *g*_*τ*_*B*_*τ*_, and study the effects of a uniform *h* on the ground state phase diagram of the system. Our CAS system is schematically shown in Fig. 1.

The Hamiltonian in Eq. (1) possesses the translational symmetry of the 2D lattice with the translational vector $a\hat{x}+2a\hat{y}$ as well as the rotational U(1) symmetry. Spontaneously breaking of these symmetries by varying the model parameters, causes the system to experience various first- and second-order phase transitions. Different diagonal and off-diagonal long-range orders appear in the ground state phase diagram of the above model which will be discussed in next sections.

### Bosonic counterpart of the CAS chains

In bosonic language, the systems of coupled uniform spin-1/2 chains are equivalent to the systems of coupled 1D optical lattices, containing hard-core bosons. These systems possess different superfluid and solid phases^76^, in the presence of intra-chains hoppings, repulsive intra-chains and attractive inter-chains interactions, but no supersolid phase is formed due to the hard-core nature of the bosons.

The three-body-constrained bosons (or semi-hard-core bosons) with the nilpotency condition ${({b}_{i}^{\dagger })}^{3}=0$, may remove this problem. This nilpotency condition signifies that one can put up two *b* particles on each lattice site. Recent studies on the coupled 1D optical lattices containing two kinds of boson, a hard-core and a semi-hard-core boson, show that the system displays different MIs and SF orders^77^, in the presence of intra-chains hoppings and inter-chains interactions, but still solid and supersolid phases are absent in the ground state phase diagram. Indeed in the absence of intra-chains repulsive interactions the translational symmetry of the system preserves and consequently no solidity occurs in the system. So, considering repulsive intra-chains interactions on the semi-hard-core bosons can result in a supersolid order in the system of coupled 1D optical lattices.

Here, by using the relations between semi-hard-core bosons and spin-1 operators^70^, we map our CAS model in Eq. (1) to a bosonic system of coupled alternating hard-core and semi-hard-core bosonic lattices. We demonstrate that the presence of repulsive intra-chains interactions is sufficient for the appearance of solid and supersolid phases.

Let us consider a system of coupled alternating 1D optical lattices with hard-core and semi-hard-core bosons, *a* and *b*, which interact via the following Hamiltonian:

3 $$\begin{array}{rcl}H & = & {H}_{a}+{H}_{b}+{H}_{ab},\\ {H}_{a} & = & \sum _{\langle i,j\rangle }[({t}^{a}{a}_{i}^{\dagger }{a}_{j}+h\mathrm{.}c\mathrm{.)}+{V}^{a}{n}_{i}^{a}{n}_{j}^{a}]-\sum _{i}{\mu }^{a}{n}_{i}^{a},\\ {H}_{b} & = & \sum _{\langle i,j\rangle }[({t}^{b}{b}_{i}^{\dagger }{b}_{j}+h\mathrm{.}c\mathrm{.)}+{V}^{b}{n}_{i}^{b}{n}_{j}^{b}]-\sum _{i}{\mu }^{b}{n}_{i}^{b},\\ {H}_{ab} & = & \sum _{\langle i,j\rangle }[({t}^{ab}{a}_{i}^{\dagger }{b}_{j}+h\mathrm{.}c\mathrm{.)}+{V}^{ab}{n}_{i}^{a}{n}_{j}^{b}]+{V}_{2}\sum _{\langle \langle i,j\rangle \rangle }{n}_{i}^{a}{n}_{j}^{b},\end{array}$$

where *H*_*a*_ (*H*_*b*_) contains interactions between *a* (*b*) bosons and *t*^*a*^ (*t*^*b*^), *V*^*a*^ (*V*^*b*^) and *μ*^*a*^ (*μ*^*b*^) are the hopping energy, the interaction and the chemical potential in *a* (*b*) sublattice. *H*_*ab*_ gives the interactions between *a* and *b* bosons with *t*^*ab*^ the hopping energy and *V*^*ab*^, and *V*_2_ the interaction energies. ${a}_{i}^{\dagger }$ (*a*_*i*_) and ${b}_{j}^{\dagger }$ (*b*_*j*_) are respectively the creation (annihilation) operators of *a* and *b* particles at sites *i* and *j*, on a 2D square lattice. The particles *a* are canonical hard-core bosons and satisfy the canonical commutation relations. The number of *a* bosons at site *i* is ${n}_{i}^{a}={a}_{i}^{\dagger }{a}_{i}$, and the nilpotency condition for these bosons is ${({a}_{i}^{\dagger })}^{2}=0$. The *b* particles are semi-hard-core bosons and satisfy the following statistics algebra^42,70^:

4 $$\begin{array}{c}[{b}_{i},{b}_{j}]=[{b}_{i}^{{{\dagger }}},{b}_{j}^{\dagger }]=0\\ \left[{b}_{i},{b}_{j}^{\dagger }\right]={\delta }_{ij}(1-{n}_{i}^{b}),[{n}_{i}^{b},{b}_{j}^{\dagger }]={\delta }_{ij}{b}_{j}^{\dagger },\end{array}$$

where ${n}_{i}^{b}$ is the number of *b* bosons which possesses the relation ${({n}_{i}^{b})}^{\dagger }={n}_{i}^{b}$.

In order to obtain the bosonic Hamiltonian in Eq. (3) we have used the following linear spin-boson transformations between *a* bosons and spin-$\frac{1}{2}$^78^, and between *b* bosons and the spin-1 operators^42^:

5 $$\begin{array}{c}{\sigma }_{i}^{z}={n}_{i}^{a}-\frac{1}{2},\,{\sigma }_{i}^{+}={a}_{i}^{\dagger },\,{\sigma }_{i}^{-}={a}_{i},\\ {\tau }_{j}^{z}={n}_{j}^{b}-\mathrm{1,}\,{\tau }_{j}^{+}=\sqrt{2}{b}_{j}^{\dagger },\,{\tau }_{j}^{-}=\sqrt{2}{b}_{j}\mathrm{.}\end{array}$$

Since these spin-boson transformations are isomorphic, all symmetries and physical properties of the CAS system (1) and the bosonic system (3) are identically the same. The bosonic Hamiltonian in Eq. (3) is transformed to the mixed-spin Hamiltonian in Eq. (1) by defining the following relations:

6 $$\begin{array}{c}{V}^{a(b)}={V}^{ab}={V}_{1},\\ {t}^{a}\to J,\,\,{t}^{b}\to J\mathrm{/2,\ }{t}^{ab}\to J/\sqrt{2},\\ {\mu }^{a}\to h+4{V}_{1}+4{V}_{2},\,\,{\mu }^{b}\to h+5{V}_{1}+2{V}_{2}\mathrm{.}\end{array}$$

## Ground State Phase Diagram

In order to obtain the ground state phase diagram of the CAS model, first we use a MF approximation to investigate the system classically and then using LSWT we take into account the effects of quantum fluctuations around MF ground state. Moreover, utilizing a generalized CMFT we obtain the modified ground state phase diagram of the CAS model.

In MF approximation, we first divide the system into four sublattices, the subsystem with spins *σ* into A and B, and the subsystem with spins *τ* into C and D, and then approximate the local spins’ averages with MF order parameters. The four-sublattice structure is expected to be emerged due to the NN and NNN interactions. By defining the MF order parameters: $\langle {\sigma }_{{i}_{A}}^{\alpha }\rangle ={m}_{A}^{\alpha }$, $\langle {\sigma }_{{i}_{B}}^{\alpha }\rangle ={m}_{B}^{\alpha }$, $\langle {\tau }_{{i}_{C}}^{\alpha }\rangle ={M}_{C}^{\alpha }$, and $\langle {\tau }_{{i}_{D}}^{\alpha }\rangle ={M}_{D}^{\alpha }$, where 〈…〉 denotes the expectation value on the MF ground state, the Hamiltonian in Eq. (1) is readily simplified to a single particle MF Hamiltonian (see Eq. 11). The expectation values of spin operators on the ground state of the MF Hamiltonian (sublattices’ magnetizations) are given in terms of other MF order parameters (see Sec. 5.1 for detail). Various kinds of long-range diagonal and off-diagonal orders are defined by these magnetizations. In the Table 1, we have defined all possible phases, appearing in the MF ground state phase diagrams.

**Table 1:** Definitions of various orders. *M*_*v*_ is the total transverse magnetization. The fillings (average number of bosons in each unit cell) are mentioned in the parentheses.

Phases	Sublattices’ magnetizations	*M*_*v*_
ST(3/6)	${m}_{A}^{z}=-\,{m}_{B}^{z}$, ${M}_{C}^{z}=-\,{M}_{D}^{z}$	0
*b*ST(4/6)	${m}_{A}^{z}={m}_{B}^{z}$, ${M}_{C}^{z}=-\,{M}_{D}^{z}$	0
MI(4/6)	${m}_{A}^{z}={m}_{B}^{z}$, ${M}_{C}^{z}={M}_{D}^{z}$	0
*a*ST(5/6)	${m}_{A}^{z}=-\,{m}_{B}^{z}$, ${M}_{C}^{z}={M}_{D}^{z}$	0
Full	${m}_{A}^{z}={m}_{B}^{z}=\mathrm{1/2}$, ${M}_{C}^{z}={M}_{D}^{z}=1$	0
STS	${m}_{A}^{z}\ne {m}_{B}^{z}\ne {M}_{C}^{z}\ne {M}_{D}^{z}$	≠0
SF	${m}_{A}^{z}={m}_{B}^{z}$, ${M}_{C}^{z}={M}_{D}^{z}$	≠0
SCF	${m}_{A}^{z}={m}_{B}^{z}$, ${m}_{A}^{x,y}=-\,{m}_{B}^{x,y}\ne 0$, ${M}_{C}^{z}={M}_{D}^{z}$, ${M}_{C}^{x,y}=-\,{M}_{D}^{x,y}\ne 0$	0

**Figure 2:**
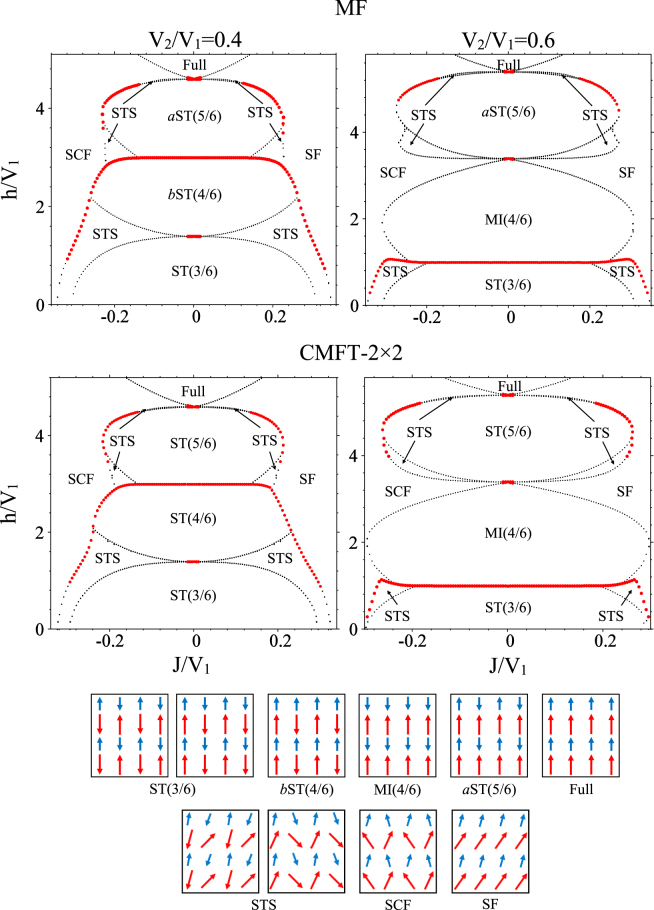
Ground state phase diagrams of the CAS system for the two different strengths of frustration: $$\frac{{V}_{2}}{{V}_{1}}=0.4$$ and $$\frac{{V}_{2}}{{V}_{1}}=0.6$$. Top and middle: MF and CMFT phase diagrams. The red (black) dotted lines show first-order (second-order) phase transitions. Bottom: schematic illustrations of various solids, SF, SCF and STS phases. The spins alignment in the left (right) panel of ST(3/6) phase occurs for *V*_2_/*V*_1_ < 0.5 ($$ > 0.5$$) to satisfy the interaction *V*_1_ (*V*_2_). Moreover, the spins alignment in the left (right) panel of the STS phase forms around the left (right) side of the ST(3/6) phase.

We have plotted in Fig. 2-top the MF ground state phase diagrams of the CAS system, for the two different strengths of frustration: *V*_2_/*V*_1_ = 0.4 and 0.6. We have also illustrated the schematic pictures of various orders, at the bottom of Fig. 2. For small values of *J*/*V*_1_, independent of the strengths of frustrations, the ground state phase diagram is symmetric with respect to the *J* = 0 line. Far from *J* = 0 line, the system, however, behaves differently for $J > 0$ and *J* < 0 regions. For large values of $J > 0$ the U(1) symmetry of the system breaks spontaneously, and the SF long range order emerges in the system where each boson is spread out over the entire lattice, with long-range phase coherence. For *J* < 0, instead of SF phase, the SCF phase appears in the phase diagram, where the transverse components of the spins lie in opposite directions (see Fig. 2, the schematic picture of SCF). This phase is characterized by a transverse staggered magnetization and a longitudinal magnetization^79^. Although SCF is not a superfluid, but as we will show by means of LSWT, its excitation spectrum is identically the same as the SF phase. In this phase due to the transverse staggered magnetization the easy plane U(1) symmetry reduces to the *Z*_2_ one^80^ and the translational symmetry is also broken. But, it is not a kind of solid since this phase possesses a gapless excitation and the longitudinal staggered magnetization is zero. This phase is not seen in the ground state phase diagram of the 2D mixed-spin system with staggered arrangement of spin-1/2 and spin-1^70^. In the staggered 2D mixed-spin system the ground state phase diagram is completely symmetric with respect to *J* = 0.

By decreasing |*J*|, at small magnetic field, aside from the U(1) symmetry (which is completely broken in $J > 0$ region and is decreased to the *Z*_2_ symmetry in *J* < 0 one), the translational symmetry of the system also breaks and a phase transition occurs from SF and SCF to the STS phase in which both diagonal and off-diagonal long range orders coexist in the system. The STS-SF and STS-SCF phase transitions are of first- or second-order which are attributed to the behavior of the low energy spin wave excitations. Indeed, the abrupt and smooth changes of the low energy excitations close to a transition point results in the discontinuous and continuous variations of the diagonal and off-diagonal order parameters.

**Figure 3:**
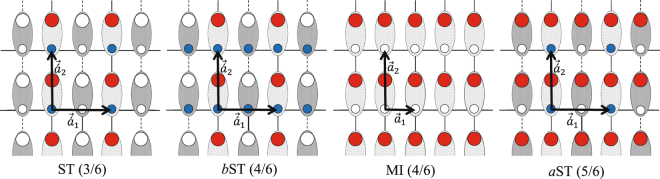
Bosonic version of solids and Mott insulator. The small (large) circles show spin *σ* = 1/2 (*τ* = 1) and filled (empty) circles show presence (absence) of particles. Each unit cell contains two sites. In symmetry preserved MI(4/6) phase, the system consists of two sublattices and the primitive vectors are ${\overrightarrow{a}}_{1}$ and ${\overrightarrow{a}}_{2}$. In the symmetry breaking ST(3/6), *b*ST(4/6) and *a*ST(5/6) phases, the system consists of four sublattices and the primitive vectors are ${{\overrightarrow{a}}^{\text{'}}}_{1}=2{\overrightarrow{a}}_{1}$ and ${{\overrightarrow{a}}^{\text{'}}}_{2}={\overrightarrow{a}}_{2}$.

In addition to the SF, SCF and STS phases, various kinds of stripe solids: ST(3/6), *b*ST(4/6) and *a*ST(5/6), respectively with fillings 3/6, 4/6, and 5/6 also appear in the phase diagram of the CAS system. The schematic pictures of these phases are plotted in the bottom of Fig. 2. Moreover, in order to show the fillings of these phases we have plotted in Fig. 3 the bosonic version of them.

In these solid phases, depending on the values of magnetic field, the translational symmetries of both subsystems or one of them break spontaneously, (in *b*ST(4/6) the translational symmetry of the subsystem with spin *τ* and in *a*ST(5/6) the translational symmetry of the subsystem with spin *σ*). The stripe solid orders with fillings 4/6 and 5/6 are the characteristics of our mixed-spin CAS system and are not seen in the phase diagram of uniform XXZ spin-1/2 models. For *V*_2_/*V*_1_ = 0.6, around *J* = 0, instead of *b*ST(4/6), the system prefers to be in the MI(4/6) phase at moderate magnetic field where both the translational and U(1) symmetries are preserved in the system. Actually, for larger values of *V*_2_/*V*_1_, the *V*_2_ interactions try to make the spins *τ* and *σ* antiparallel, such that the translational symmetry of both subsystems preserves at moderate magnetic field. This behavior that the translational symmetry does not break even at large interactions, is the characteristic of the two-component systems with inter-components interaction, which has also been seen in the staggered mixed-spin system at *V*_2_/*V*_1_ < 0.5^70^.

In order to obtain the more accurate phase diagrams, we employ CMFT with different cluster sizes. CMFT is an extension of the standard MF approximation in which clusters of multiple sites are used as an approximate system instead of single sites^22,33,70,81^. Recently we have generalized CMFT for the staggered 2D mixed-spin system^70^. Treating exactly the interactions within the cluster and including the interaction of spins outside the cluster as an effective field, one can partially take into account fluctuations around classical ground state as well as the effects of correlations of particles. Similar to our MF analysis, we assume a background with four-sublattice structure and embed a cluster of *N*_*C*_ sites into this background. Instead of treating the many-body problem in the whole system, we consider the following effective cluster Hamiltonian:

7 $${H}_{C}^{eff}={H}_{C}+{H}_{\bar{C}},$$

where the interactions within clusters are given by *H*_*C*_ while the interactions of spins inside the clusters with the rest of the system are included in ${H}_{\bar{c}}$. The Hamiltonian *H*_*C*_ is given by Eq. (1) where the summations run over sites *i*, *j* ∈ *C*, and the Hamiltonian ${H}_{\bar{c}}$ is given in terms of the effective fields ${\overrightarrow{h}}_{i}^{eff}$ and ${\overrightarrow{g}}_{i}^{eff}$ as:

8 $${H}_{\bar{C}}=\sum _{i\in C}({\overrightarrow{h}}_{i}^{eff}\cdot {\overrightarrow{\sigma }}_{i}+{\overrightarrow{g}}_{i}^{eff}\cdot {\overrightarrow{\tau }}_{i}\mathrm{).}$$

These effective fields are given in terms of CMFT ground state magnetizations, which are computed self-consistently (see Sec. 5.2 for detail).

In Fig. 2-middle we have plotted CMFT phase diagrams for the two different strengths of frustration: *V*_2_/*V*_1_ = 0.4 and 0.6, using cluster of four sites [CMFT-(2 × 2)]. Comparison between MF and CMFT phase diagrams shows that, due to the effects of quantum fluctuations the *b*ST(4/6) and *a* ST(5/6) solid phases respectively convert to the ST(4/6) and ST(5/6) ones in which the translational symmetry of both subsystems break. It is also seen that except for the STS-SF transition lines there is no considerable changes in the MF phase diagrams in the presence of quantum fluctuations. The slight deviations of the STS-SF transition lines, for ${V}_{2}/{V}_{1} > 0.5$, at large magnetic field, are attributed to the large amount of quantum fluctuations at these boarders. In order to see the behavior of quantum fluctuations in each phases, we utilize LSWT and study the variations of spin waves’ number in all sublattices. Using Holstein-Primakoff (HP) transformations (see Eqs (17) and (18)) in Sec. 5.3), the Hamiltonian in Eq. (1) transforms to the following spin wave Hamiltonian:

9 $$\tilde{H}={E}_{0}+\sum _{{\rm{k}}}{\psi }_{{\rm{k}}}^{\dagger }{H}_{{\rm{k}}}{\psi }_{{\rm{k}}},$$

where *E*_0_ is the classical MF energy, *H*_k_ is a square matrix in Fourier space, consisting the coefficients of bilinear terms, and *ψ*_k_ is a vector in terms of HP bosonic creation and annihilation operators. Dimensions of *ψ*_k_ and *H*_k_ depend on the number of sublattices in the MF ordered phases (see Sec. 5.3 for detail). Paraunitary diagonalization^82^ of *H*_k_ yields the excitation spectra in each phase, as well as the HP bosons’ number.

**Figure 4:**
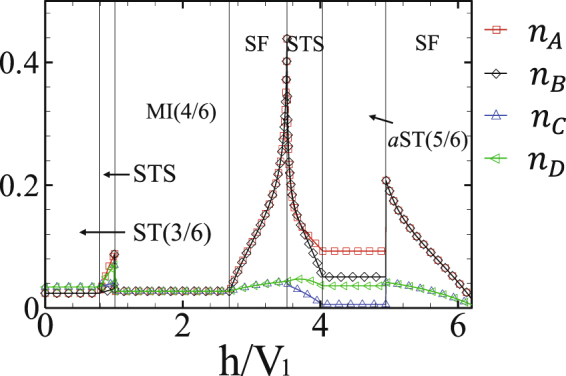
Number of HP bosons on the MF ground state for $\frac{{V}_{2}}{{V}_{1}}=0.6$ and $\frac{J}{{V}_{1}}=0.24$. *n*_*A*_ and *n*_*B*_ are the amount of quantum fluctuations in the subsystem with spin *σ*, and *n*_*C*_ and *n*_*D*_ are in the subsystem with spin *τ*. Overall quantum fluctuations are not strong enough to destroy MF orders, but for larger values of NNN interactions, the STS-SF transition lines are modified in comparison with the corresponding MF results.

Amount of quantum fluctuations in different phases is given by HP bosons’ numbers *n*_*A*_, *n*_*B*_, *n*_*C*_ and *n*_*D*_, respectively in the sublattices *A*, *B*, *C* and *D*. As it is seen from Fig. 4, in our mixed-spin model overall quantum fluctuations are not strong enough to destroy the MF orders and the MF predictions are reliable. However, at the second-order SF-STS and first-order SF-*a*ST(5/6) transition lines, they are not negligible and we should take them into account for reaching to the accurate ground state phase diagrams. Also, since *n*_*A*_(*n*_*C*_) is not equal to *n*_*B*_(*n*_*D*_), the *b*ST(4/6) and *a*ST(5/6) solids convert to the ST(4/6) and ST(5/6) ones, respectively (see Fig. 2, CMFT-(2 × 2) phase diagram). It should be noticed that although quantum fluctuations break the translational symmetry of both subsystems but the fillings do not change. Moreover, in the presence of quantum fluctuations, part of the second-order STS-SF transition line below the ST(5/6) phase transforms to a first order one. This means that the MF prediction concerning the kind of transition order at this region is not correct, and more precise approaches should be employed to obtain the STS-SF critical and tricritical points.

**Figure 5:**
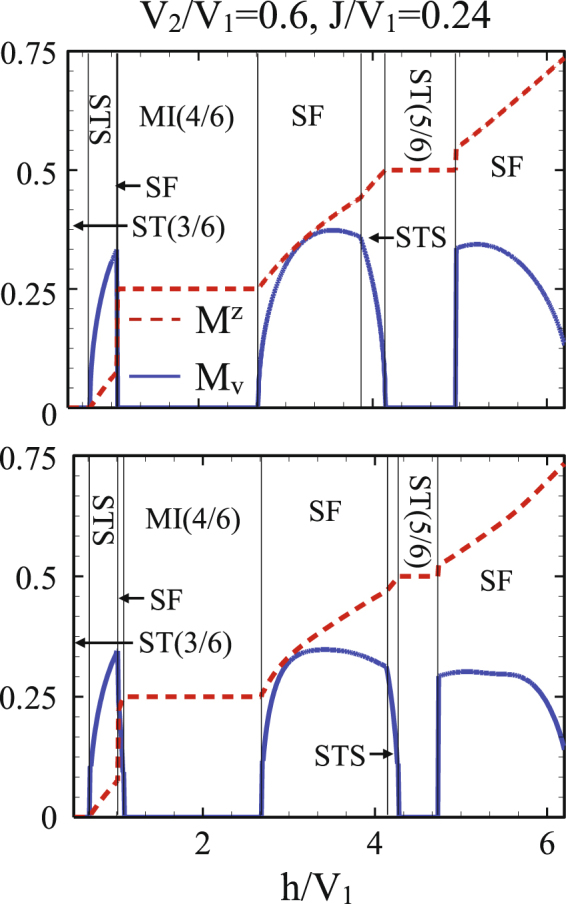
Diagonal and off-diagonal order parameters, computed by CMFT-(2 × 2) (top) and CMFT-(2 × 4) (bottom) for $\frac{{V}_{2}}{{V}_{1}}=0.6$ and $\frac{J}{{V}_{1}}=0.24$.

We also investigate the behavior of both diagonal and off-diagonal order parameters considering clusters with larger sizes in CMFT. Employing clusters of eight spins [CMFT-(2 × 4)], we have computed the sublattices longitudinal and transverse magnetizations for different values of *h* and *J* (see Fig. 5). Since quantum fluctuations are strong around first order transition lines, some modifications around these lines are expected. These are clearly seen by comparison of the phases boarders of CMFT-(2 × 2) and CMFT-(2 × 4) in Fig. 5. The behavior of the order parameters indicates that the STS phase around ST(5/6) solid becomes narrower in the presence of quantum fluctuations. It seems that these regions tend to be disappeared when we use CMFT with larger cluster sizes. The STS phase appeared at small magnetic fields is however stable. The instability of STS phase at larger magnetic field can be explained as follows. The appearance of the STS phase is in fact the result of the competition between the staggered magnetization along *z* direction (diagonal order) and the total transverse magnetization (off-diagonal order). At large magnetic field, in the presence of quantum fluctuations, both order parameters decrease but the staggered magnetization is more sensitive and diminishes around the phase boarder. So the STS region at larger magnetic field is decreased in CMFT-(2 × 4).

The CMFT becomes exact when the cluster size goes to infinity. In practice, we are faced with computational limitations due to the increasing of clusters’ sizes and can not consider clusters of large sizes. We thus should employ other techniques such as quantum Monte Carlo simulations to obtain the exact phase diagram of the CAS model, at large magnetic field.

**Figure 6:**
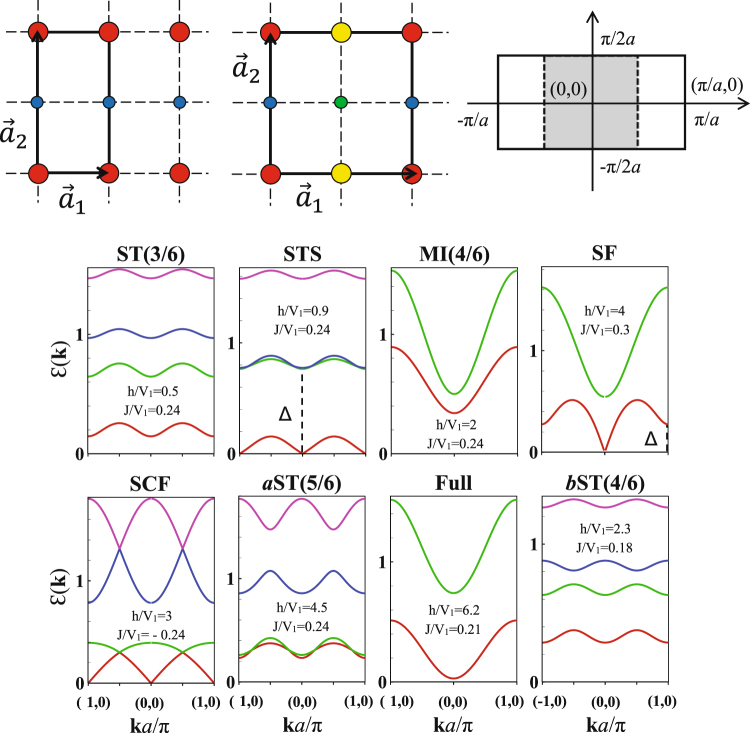
Spin wave excitation spectra in various phases of the CAS system. Number of excitation modes reflects the number of sublattices in each phase. Top-left: 2D lattice with primitive vectors ${\overrightarrow{a}}_{1}=a\hat{x}$ and ${\overrightarrow{a}}_{2}=2a\hat{y}$ for the MI(4/6), Full and SF phases where the original lattice symmetry is preserved. Top-center: 2D lattice with primitive vectors ${\overrightarrow{a}}_{1}=2a\hat{x}$ and ${\overrightarrow{a}}_{2}=2a\hat{y}$ for the solid, supersolid and SCF phases where the original lattice symmetry in *x* direction is broken. Top-right: the unfolded and folded Brillouin zones. Middle and bottom: spin wave excitations of all phases in *k*_*y*_ = 0 direction. The parameter Δ in the SF and STS supersolid phases is the roton energy gap. In solid phases all excitations are gapped and the lowest spectrum has quadratic dispersion (*k*^2^) around **k** = (0, 0). Whereas, in STS, SF and SCF phases, a gapless Goldstone mode appears in the excitation spectra. All plots are for *V*_2_/*V*_1_ = 0.6 except *b*ST(4/6) which is for *V*_2_/*V*_1_ = 0.4.

We have also plotted in Fig. 6, the spin wave excitation spectra in all phases of the CAS system. Number of excitation modes and their behavior depend on the number of sublattices as well as their longitudinal and transverse magnetizations. According to the translational symmetry of the CAS system, the primitive vectors in the SF and MI phases are ${\overrightarrow{a}}_{1}=a\hat{x}$ and ${\overrightarrow{a}}_{2}=a\hat{y}$, and there are two excitation modes in the system. However, when the translational symmetry breaks in *x* direction, as in the different solids, STS and SCF phases, the primitive vectors are ${\overrightarrow{a}}_{1}=2a\hat{x}$ and ${\overrightarrow{a}}_{2}=2a\hat{y}$ and the first Brillouin zone is folded in *x* direction (see Fig. 6-top). In these phases there exist four excitation modes in the system.

In STS and SF phases, as a result of the continues U(1) symmetry breaking, a gapless Goldstone mode with a roton-like minimum Δ appears in the excitation spectra (see Fig. 6). The slope of the line connecting this roton minimum to the origin in the critical velocity of the superfluid. The critical velocity in the STS phase depends inversely on the values of magnetic field. The critical velocity of the STS at lower magnetic field is larger than the one in the higher field. For *J* < 0, in SCF phase, the spins in each subsystem are antiparallel in *xy* plane. In this phase the translational symmetry breaks which results in a non-zero transverse staggered magnetization. Due to the translational symmetry breaking, four excitation modes appear in the energy spectrum. Moreover, since the U(1) symmetry also decreases to *Z*_2_ one in this phase the low energy excitation is gapless with linear dispersion around **k** = (0, 0), and the roton minimum is folded back to the origin.

## Anisotropic CAS Models

In the previous section we obtained the ground state phase diagram of the isotropic CAS model in which the inter-chains and intra-chains NN interactions (*V*_1_) and hopping energies (*J*) were the same. In this section we consider two anisotropic CAS models: (*i*) a CAS system with different inter- and intra-chains NN interactions, and (*ii*) a CAS system with different inter- and intra-chains hopping energies, and investigate the effects of these anisotropies on the stability of the STS order appeared in the ground state phase diagram of the system. Following, we study these two anisotropic systems, separately.

### Anisotropic CAS with different NN interactions

Let us consider the intra-chains and inter-chains NN interactions to be respectively *V*_1_ and ${V}_{1}^{\text{'}}$.

**Figure 7:**
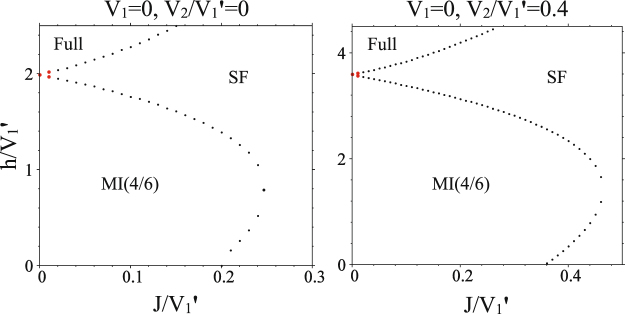
CMFT *J*−*h* ground state phase diagrams of the anisotropic CAS model in the absence of the intra-chains interactions, (*V*_1_ = 0). Independent of the values of NNN interactions, no supersolid order appears in the phase diagram.

In the absence of intra-chains interaction, at *V*_1_ = 0, each 1D lattices are described by a XY Hamiltonian and no supersolid order appears in the ground state phase diagram of the system. In this case, when the inter-chains interactions are attractive (${V}_{1}=\mathrm{0,}\,{V}_{1}^{\text{'}} < 0$), at *h* = 0 the system is in the ST(3/6) phase and a phase transition occurs to the SF phase at $J/|{V}_{1}^{\text{'}}|\,\approx \,0.23$. In the presence of magnetic field, the system enters the Full phase (not shown). When the inter-chains interactions are repulsive (${V}_{1}=\mathrm{0,}\,{V}_{1}^{\text{'}} > 0$) the system displays MI(4/6), in addition to the SF order, but no supersolidity occurs in the phase diagram of the model (see Fig. 7). This is due the fact that in the absence of intra-chains interactions, the translational symmetry of the chains preserves and the system is always MI(4/6) or SF, below the saturation field. It should be noticed that the MI(4/6) phase, appeared in the ground state phase diagram of the isotropic CAS model (see Fig. 2), is a result of competition between NNN interaction and magnetic field. However, in the mentioned anisotropic CAS model this phase emerges at *h* = *V*_2_ = 0 where the intra-chains interactions are absent (see left panel of Fig. 7).

**Figure 8:**
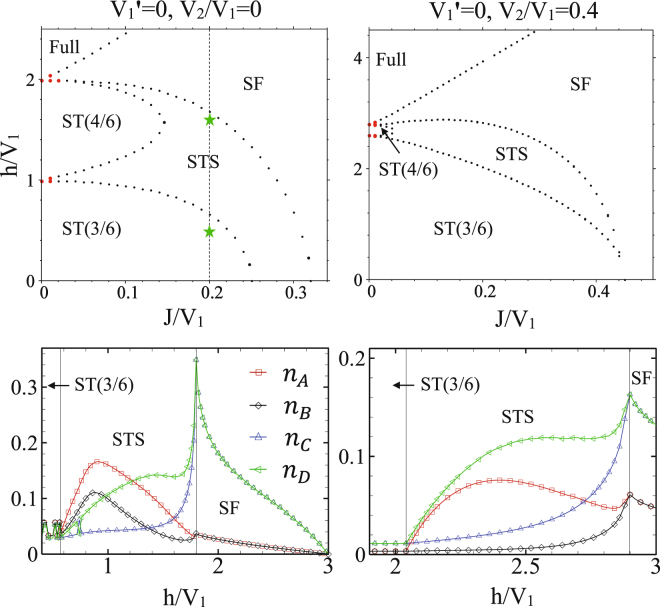
Top: CMFT *J*−*h* ground state phase diagrams of the anisotropic CAS model in the absence of the inter-chains interactions, (${V}_{1}^{\text{'}}$ = 0). Top-left: in the absence of NNN interactions, (*V*_2_ = 0). Top-right: NNN interactions are *V*_2_/*V*_1_ = 0.4. Bottom: Quantum fluctuations (HP bosons’ number) on MF ground state, at the line *J*/*V*_1_ = 0.2, without (bottom-left) and with (bottom-right) NNN interaction. Except for near $J\simeq 0$, the rest of phase transitions are of second order. Green stars are the SF-STS and STS-ST(3/6) critical points, computed by CMFT-(2 × 4).

In the absence of inter-chains NN interactions, at ${V}_{1}^{\text{'}}=0$, the anisotropic CAS system is composed of coupled 1D spin-1 and spin-1/2 XXZ models in a longitudinal magnetic field. In this system, when the intra-chains interactions are attractive (${V}_{1}^{\text{'}}$ = 0, *V*_1_ < 0), at *h* = 0 the system is in the MI(4/6) phase and there is a phase transition to the SF order at *J*/|*V*_1_| ≈ 0.26, and in the presence of magnetic field, at *h* ≠ 0, trivially the system is in the fully polarized phase (not shown). However, when the intra-chains interactions are repulsive (${V}_{1}^{\text{'}}=\mathrm{0,}\,{V}_{1} > 0$), due to the breaking of the translational and U(1) symmetries, independent of *V*_2_, the system exhibits the STS phase. In addition to the STS phase, the ST(3/6) and ST(4/6) solids, and also SF orders also appear in the phase diagram of the system (see upper panels of Fig. 8). Comparison of the phase diagrams of *V*_2_ = 0 and *V*_2_ ≠ 0 in Fig. 8 shows that the presence of the NNN interactions *V*_2_ decreases the STS and ST(4/6) regions. It is surprising that in the absence of *V*_2_ the STS phase emerges in the phase diagram even at zero magnetic field. The amount of quantum fluctuations in this anisotropic CAS system is plotted in Fig. 8 (lower panels). As it is seen the fluctuations in STS phase are not strong and we expect the STS phases appeared in the CMFT phase diagrams to be stable in the presence of quantum fluctuations. This achievement is also confirmed by our CMFT-(2 × 4) results. According to our CMFT-(2 × 4) data (not shown), for ${V}_{1}^{\text{'}}$ = *V*_2_ = 0 although the SF-STS and STS-ST(3/6) critical points shift to the lower values of magnetic fields, but the STS region does not become narrower (see green stars in Fig. 8).

**Figure 9:**
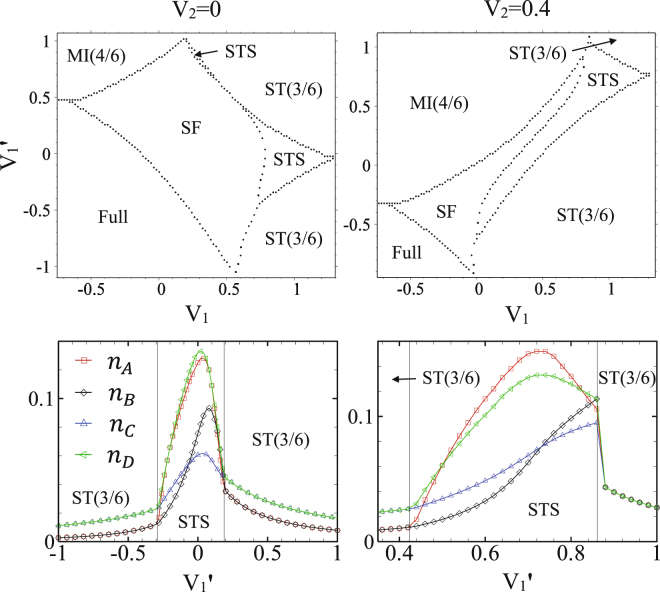
Top: CMFT *V*_1_−${V}_{1}^{\text{'}}$ ground state phase diagrams of the anisotropic CAS model for *h* = 1 and *J* = 0.2. Bottom: amount of quantum fluctuations at line *V*_1_ = 0.9 (left), and *V*_1_ = 0.8 (right). All transitions are of second order.

Above results indicate that, the presence of finite repulsive intra-chains interactions are necessary for the STS and ST phases to be emerged in the anisotropic CAS phase diagram. To check this idea we have also plotted in Fig. 9 the *V*_1_−${V}_{1}^{\text{'}}$ phase diagram of the anisotropic CAS system, for the two different values of NNN interactions: *V*_2_ = 0, and 0.4 at *h* = 1 and *J* = 0.2. These figures show that in the presence of attractive intra-chains interactions, the two Mott insulating phases, MI(4/6) and Full, also appear in the phase diagram in addition to the SF order. However, there is no signature of STS and ST phases in this region in both values of NNN interaction. The STS and ST phases emerge in the *V*_1_ > 0 region, independent of the strengths of ${V}_{1}^{\text{'}}$ and *V*_2_, where the translational symmetry breaks in the presence of repulsive intra-chains interactions. The small amount of quantum fluctuations in these regions is the reason of the stability of all orders.

From Fig. 9 it is also seen that in the isotropic CAS model (${V}_{1}={V}_{1}^{\text{'}}$) it is impossible to find the STS phase in the absence of NNN interactions. Previous studies on the 2D Bose-Hubbard model with three-body-constrained bosons, show solid and superfluid phases in the presence of isotropic NN interactions and hopping terms^83^, but no supersolidity occurs in this system. Actually, NNN interactions can stabilize the supersolid phases in this model.

### Anisotropic CAS with different hoppings

**Figure 10:**
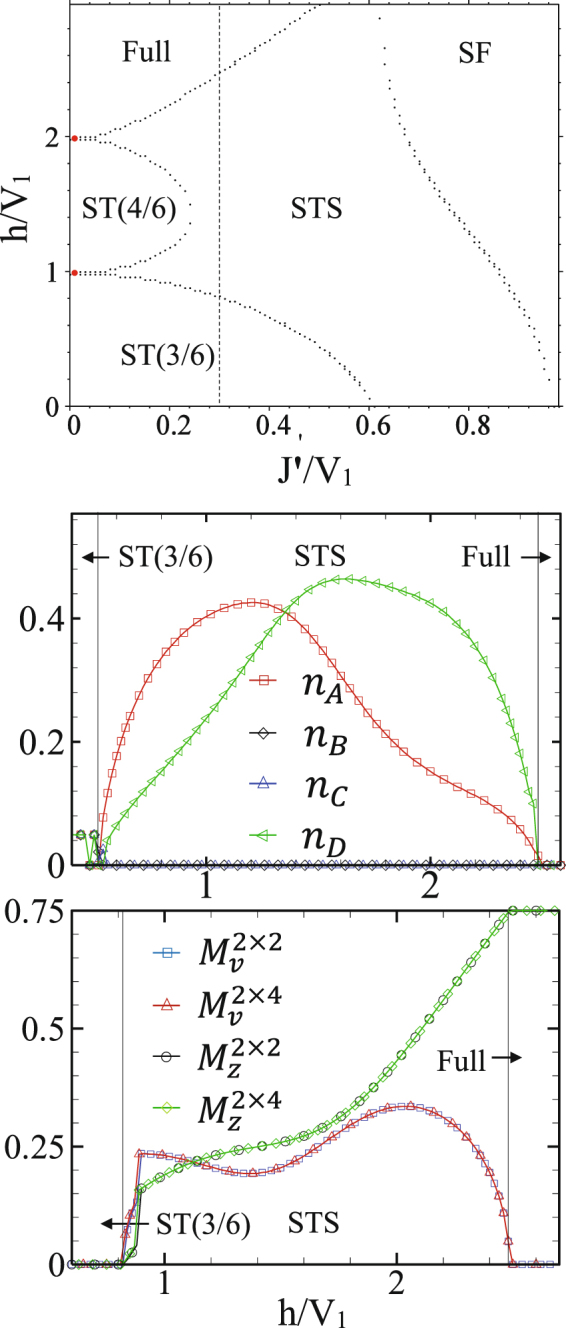
Top: CMFT *J*′−*h* ground state phase diagram of the anisotropic CAS model (${V}_{1}^{\text{'}}$ = *V*_2_ = 0). Middle: amount of quantum fluctuations at line *J*′/*V*_1_ = 0.3, shown in the phase diagram. Bottom: Order parameters computed by CMFT-(2 × 2) and CMFT-(2 × 4) at the same line.

In this subsection we consider an anisotropic CAS model with different inter- and intra-chains hopping energies and investigate the effects of this anisotropy on the ground state phase diagram of the system. Let us consider the intra- and inter-chains hopping energies to be respectively *J* and *J*′. Suppose one of the hopping energies *J* or *J*′ to be zero. Our CMFT results show that the presence of inter-chains hoppings together with the repulsive intra-chains NN interactions are sufficient for the appearance of STS phase (see Fig. 10). The behavior of HP bosons’ number shows that in the STS phase the quantum fluctuations are exactly zero in the sublattices B and C, but considerable in A and D. This means that we should expect some modifications on the sublattices’ magnetizations in the presence of fluctuations. However, surprisingly we see that the order parameters computed by CMFT-(2 × 2) and -(2 × 4) are exactly the same (see Fig. 10) which is an indication of the stability of the STS phase in this system.

### Minimal model

Based on the above results we conclude that, in the absence of inter-chains hoppings, the minimal mixed-spin CAS model for the supersolidity is given by the following Hamiltonian:

10 $$H={H}_{XXZ}^{\sigma }+{H}_{XXZ}^{\tau }+{H}_{Z}^{\sigma \tau },$$

where, ${H}_{XXZ}^{\sigma }$ and ${H}_{XXZ}^{\tau }$ are respectively the spin-1/2 and spin-1 XXZ Hamiltonians. ${H}_{Z}^{\sigma \tau }$ is Ising Hamiltonian and couples the spin-1/2 and spin-1 chains. In bosonic language, the presence of off-site intra-components repulsive interactions and hopping energies together with the inter-components repulsive or attractive interactions, are sufficient to find the STS phase in the two-component hard-core Bose-Hubbard model. On the other hand, in the presence of NN inter-chains hoppings, the minimal mixed-spin CAS model for the supersolidity is, instead, spin-1 and spin-1/2 Ising chains interacting via a XY Hamiltonian. In two-component bosonic system inter-component hoppings could be possible by including different hyperfine states of the same atoms. So, in the case of possible inter-component hoppings, repulsive off-site intra-component interactions are necessary to find the STS phase.

## Methods

In this section we explain the details of MF, CMFT and LSWT.

### Mean field approximation

In the MF approach, we approximate local spins’ averages with MF order parameters. By defining the following MF order parameters: $\langle {\sigma }_{{i}_{A}}^{x,y,z}\rangle ={m}_{A}^{x,y,z}$, $\langle {\sigma }_{{i}_{B}}^{x,y,z}\rangle ={m}_{B}^{x,y,z}$, $\langle {\tau }_{{i}_{C}}^{x,y,z}\rangle ={M}_{C}^{x,y,z}$, and $\langle {\tau }_{{i}_{D}}^{x,y,z}\rangle ={M}_{D}^{x,y,z}$, where 〈…〉 denotes the expectation value on the MF ground state, the Hamiltonian in Eq. (1) is readily simplified to the MF Hamiltonian:

11 $${H}^{MF}={H}_{A}^{MF}+{H}_{B}^{MF}+{H}_{C}^{MF}+{H}_{D}^{MF},$$

with

12 $$\begin{array}{c}\begin{array}{rcl}{H}_{A}^{MF} & = & \sum _{i\in A}[\,-4J({m}_{B}^{x}+{M}_{D}^{x}){\sigma }_{i}^{x}-4J({m}_{B}^{y}+{M}_{D}^{y}){\sigma }_{i}^{y}-(h-2{V}_{1}({m}_{B}^{z}+{M}_{D}^{z})-4{V}_{2}{M}_{C}^{z}){\sigma }_{i}^{z}],\\ {H}_{B}^{MF} & = & \sum _{i\in B}[\,-\,4J({m}_{A}^{x}+{M}_{C}^{x}){\sigma }_{i}^{x}-4J({m}_{A}^{y}+{M}_{C}^{y}){\sigma }_{i}^{y}-(h-2{V}_{1}({m}_{A}^{z}+{M}_{C}^{z})-4{V}_{2}{M}_{D}^{z}){\sigma }_{i}^{z}],\\ {H}_{C}^{MF} & = & \sum _{i\in C}[\,-\,4J({m}_{B}^{x}+{M}_{D}^{x}){\tau }_{i}^{x}-4J({m}_{B}^{y}+{M}_{D}^{y}){\tau }_{i}^{y}-(h-2{V}_{1}({m}_{B}^{z}+{M}_{D}^{z})-4{V}_{2}{m}_{A}^{z}){\tau }_{i}^{z}],\\ {H}_{D}^{MF} & = & \sum _{i\in D}[\,-\,4J({m}_{A}^{x}+{M}_{C}^{x}){\tau }_{i}^{x}-4J({m}_{A}^{y}+{M}_{C}^{y}){\tau }_{i}^{y}-(h-2{V}_{1}({m}_{A}^{z}+{M}_{C}^{z})-4{V}_{2}{m}_{B}^{z}){\tau }_{i}^{z}\mathrm{].}\end{array}\end{array}$$

where *J* and *V*_1_ are NN hopping and interaction, respectively. Diagonalization of *H*^*MF*^ leads to four coupled equations for the MF order parameters. Solving these equations self-consistently gives the sublattices’ magnetizations.

### Cluster mean field theory

**Figure 11:**
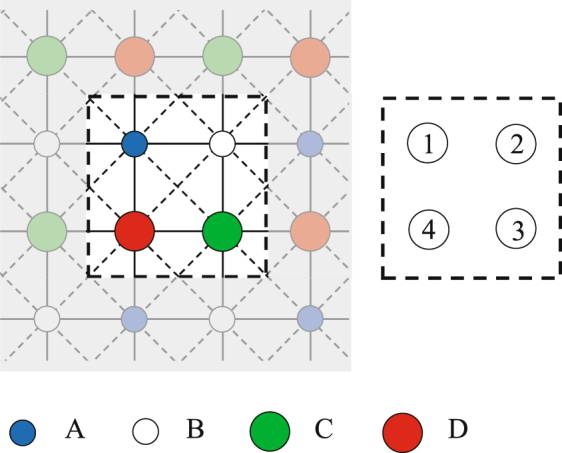
Left: a cluster with four spins. The small (large) circles show spins *σ* = 1/2 (*τ* = 1). Right: Different sites inside the cluster are labeled by numbers.

In CMFT we divide the lattice into clusters of *N*_*C*_ sites. In CMFT-(2 × 2) each cluster includes *N*_*C*_ = 4 spins, two spins 1 and two spins 1/2 (see Fig. 11). The effective magnetic fields seen by each spin inside a 2 × 2 cluster are given by:

13 $$\begin{array}{rcl}{\overrightarrow{h}}_{1}^{eff} & = & -\,2J({m}_{B}^{x}+{M}_{D}^{x})\hat{x}-2J({m}_{B}^{y}+{M}_{D}^{y})\hat{y}+[{V}_{1}({m}_{B}^{z}+{M}_{D}^{z})+3{V}_{2}{M}_{C}^{z}]\hat{z},\\ {\overrightarrow{h}}_{2}^{eff} & = & -\,2J({m}_{A}^{x}+{M}_{C}^{x})\hat{x}-2J({m}_{A}^{y}+{M}_{C}^{y})\hat{y}+[{V}_{1}({m}_{A}^{z}+{M}_{C}^{z})+3{V}_{2}{M}_{D}^{z}]\hat{z},\\ {\overrightarrow{g}}_{3}^{eff} & = & -\,2J({m}_{B}^{x}+{M}_{D}^{x})\hat{x}-2J({m}_{B}^{y}+{M}_{D}^{y})\hat{y}+[{V}_{1}({m}_{B}^{z}+{M}_{D}^{z})+3{V}_{2}{m}_{A}^{z}]\hat{z},\\ {\overrightarrow{g}}_{4}^{eff} & = & -\,2J({m}_{A}^{x}+{M}_{C}^{x})\hat{x}-2J({m}_{A}^{y}+{M}_{C}^{y})\hat{y}+[{V}_{1}({m}_{A}^{z}+{M}_{C}^{z})\hat{z}+3{V}_{2}{m}_{B}^{z}]\hat{z},\end{array}$$

where the order parameters ${m}_{A,B}^{x,y,z}$ and ${M}_{C,D}^{x,y,z}$ are the averages of spin operators on the CMFT-(2 × 2) ground state, which are computed self-consistently.

**Figure 12:**
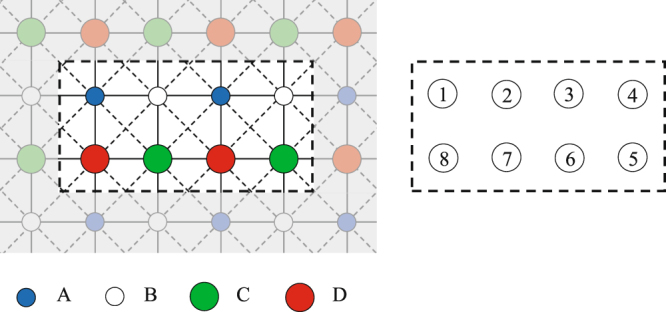
Left: a cluster with eight spins. Right: label of different sites inside the cluster.

In CMFT-(2 × 4) each cluster includes *N*_*C*_ = 8 spins, four spins 1 and four spins 1/2 (see Fig. 12). The effective magnetic fields seen by each spin inside a 2 × 4 cluster are given by:

14 $$\begin{array}{rcl}{\overrightarrow{h}}_{1}^{eff} & = & -\,2J({m}_{B}^{x}+{M}_{D}^{x})\hat{x}-2J({m}_{B}^{y}+{M}_{D}^{y})\hat{y}+[{V}_{1}({m}_{B}^{z}+{M}_{D}^{z})+3{V}_{2}{M}_{C}^{z}]\hat{z},\\ {\overrightarrow{h}}_{2}^{eff} & = & -\,2J{M}_{C}^{x}\hat{x}-2J{M}_{C}^{y}\hat{y}+[{V}_{1}{M}_{C}^{z}+2{V}_{2}{M}_{D}^{z}]\hat{z},\\ {\overrightarrow{h}}_{3}^{eff} & = & -\,2J{M}_{D}^{x}\hat{x}-2J{M}_{D}^{y}\hat{y}+[{V}_{1}{M}_{D}^{z}+2{V}_{2}{M}_{C}^{z}]\hat{z},\\ {\overrightarrow{h}}_{4}^{eff} & = & -\,2J({m}_{A}^{x}+{M}_{C}^{x})\hat{x}-2J({m}_{A}^{y}+{M}_{C}^{y})\hat{y}+[{V}_{1}({m}_{A}^{z}+{M}_{C}^{z})+3{V}_{2}{M}_{D}^{z}]\hat{z},\\ {\overrightarrow{g}}_{5}^{eff} & = & -\,2J({m}_{B}^{x}+{M}_{D}^{x})\hat{x}-2J({m}_{B}^{y}+{M}_{D}^{y})\hat{y}+[{V}_{1}({m}_{B}^{z}+{M}_{D}^{z})+3{V}_{2}{m}_{A}^{z}]\hat{z},\\ {\overrightarrow{g}}_{6}^{eff} & = & -\,2J{m}_{A}^{x}\hat{x}-2J{m}_{A}^{y}\hat{y}+[{V}_{1}{m}_{A}^{z}+2{V}_{2}{m}_{B}^{z}]\hat{z},\\ {\overrightarrow{g}}_{7}^{eff} & = & -\,2J{m}_{B}^{x}\hat{x}-2J{m}_{B}^{y}\hat{y}+[{V}_{1}{m}_{B}^{z}+2{V}_{2}{m}_{A}^{z}]\hat{z},\\ {\overrightarrow{g}}_{8}^{eff} & = & -\,2J({m}_{A}^{x}+{M}_{C}^{x})\hat{x}-2J({m}_{A}^{y}+{M}_{C}^{y})\hat{y}+[{V}_{1}({m}_{A}^{z}+{M}_{C}^{z})+3{V}_{2}{m}_{B}^{z}]\hat{z},\end{array}$$

where the order parameters ${m}_{A,B}^{x,y,z}$ and ${M}_{C,D}^{x,y,z}$ are given by

15 $$\begin{array}{rcl}{m}_{A}^{x,y,z} & = & \frac{1}{2}(\langle {\sigma }_{1}^{x,y,z}\rangle +\langle {\sigma }_{3}^{x,y,z}\rangle ),\,{m}_{B}^{x,y,z}=\frac{1}{2}(\langle {\sigma }_{2}^{x,y,z}\rangle +\langle {\sigma }_{4}^{x,y,z}\rangle ),\\ {M}_{C}^{x,y,z} & = & \frac{1}{2}(\langle {\tau }_{5}^{x,y,z}\rangle +\langle {\tau }_{7}^{x,y,z}\rangle ),\,{M}_{D}^{x,y,z}=\frac{1}{2}(\langle {\tau }_{6}^{x,y,z}\rangle +\langle {\tau }_{8}^{x,y,z}\rangle \mathrm{).}\end{array}$$

Here, the expectation values are on the CMFT-(2×4) ground state, which are computed self-consistently.

### Linear spin wave theory

Let us perform the following local rotations:

16 $$(\begin{array}{c}{\mathop{\sigma }\limits^{ \sim }}_{i}^{x}\\ {\mathop{\sigma }\limits^{ \sim }}_{i}^{y}\\ {\mathop{\sigma }\limits^{ \sim }}_{i}^{z}\end{array})={\mathscr{R}}({\varphi }_{i},{\theta }_{i},\,0)(\begin{array}{c}{\sigma }_{i}^{x}\\ {\sigma }_{i}^{y}\\ {\sigma }_{i}^{z}\end{array}),\,\,\,(\begin{array}{c}{\mathop{\tau }\limits^{ \sim }}_{i}^{x}\\ {\mathop{\tau }\limits^{ \sim }}_{i}^{y}\\ {\mathop{\tau }\limits^{ \sim }}_{i}^{z}\end{array})={\mathscr{R}}({\phi }_{i},{\vartheta }_{i},\,0)(\begin{array}{c}{\tau }_{i}^{x}\\ {\tau }_{i}^{y}\\ {\tau }_{i}^{z}\end{array}),$$

where ${\mathcal R}$ is the rotation matrix and *ϕ*_*i*_, *θ*_*i*_, *φ*_*i*_, and *ϑ*_*i*_ are the Euler angles given by $\cos \,{\theta }_{i}=\langle {\sigma }_{i}^{z}\rangle /\sigma$, $\tan \,{{\varphi }}_{i}=\langle {\sigma }_{i}^{y}\rangle /\langle {\sigma }_{i}^{x}\rangle$, $\cos \,{\vartheta }_{j}=\langle {\tau }_{j}^{z}\rangle /\tau$ and $\tan \,{{\phi }}_{j}=\langle {\tau }_{j}^{y}\rangle /\langle {\tau }_{j}^{x}\rangle$. Here, $\langle {\sigma }_{i}^{\alpha }\rangle$ and $\langle {\tau }_{i}^{\alpha }\rangle$ are the sublattices’ magnetizations computed by means of MF theory. Using the following Holstein-Primakoff (HP) transformations:

17 $$\begin{array}{rcl}{\tilde{\sigma }}_{i}^{z} & = & \sigma -{\hat{a}}_{i}^{\dagger }{\hat{a}}_{i},\\ {\tilde{\sigma }}_{i}^{+} & = & \sqrt{2\sigma -{\hat{a}}_{i}^{\dagger }{\hat{a}}_{i}}\,{\hat{a}}_{i}\approx \sqrt{2\sigma }\,{\hat{a}}_{i},\\ {\tilde{\sigma }}_{i}^{-} & = & {\hat{a}}_{i}^{\dagger }\sqrt{2\sigma -{\hat{a}}_{i}^{\dagger }{\hat{a}}_{i}}\approx \sqrt{2\sigma }\,{\hat{a}}_{i}^{\dagger },\end{array}$$

and

18 $$\begin{array}{rcl}{\tilde{\tau }}_{j}^{z} & = & \tau -{\hat{d}}_{j}^{\dagger }{\hat{d}}_{j},\\ {\tilde{\tau }}_{j}^{+} & = & \sqrt{2\tau -{\hat{d}}_{j}^{\dagger }{\hat{d}}_{j}}\,{\hat{d}}_{j}\approx \sqrt{2\tau }\,{\hat{d}}_{j},\\ {\tilde{\tau }}_{j}^{-} & = & {\hat{d}}_{j}^{\dagger }\sqrt{2\tau -{\hat{d}}_{j}^{\dagger }{\hat{d}}_{j}}\approx \sqrt{2\tau }\,{\hat{d}}_{j}^{\dagger },\end{array}$$

the Hamiltonian in Eq. (1) is expressed in terms of HP bosons as:

19 $$\tilde{H}={E}_{0}+\sum _{{\rm{k}}}{\psi }_{{\rm{k}}}^{\dagger }{H}_{{\rm{k}}}{\psi }_{{\rm{k}}},$$

where *E*_0_ is the classical MF energy, *H*_k_ is a square matrix consists of the coefficient of bilinear terms, and *ψ*_k_ is a vector in terms of HP bosonic creation and annihilation operators. The matrix *H*_k_ has the following general form:

20 $${H}_{{\rm{k}}}=(\begin{array}{cc}A & B\\ {B}^{\ast } & {A}^{\ast }\end{array}),$$

where the dimensions of A, B, *ψ*_k_ and *H*_k_ depend on the number of sublattices in the MF ordered phases. Paraunitary diagonalization^82^ of *H*_k_ yields the excitation spectra in each phase.

Now we obtain the spin wave Hamiltonian *H*_k_ and the vector *ψ*_k_ for all phases. In all solids, supersolid and SCF phases, according to the translational symmetry breaking, the classical background has a four-sublattice structure and four HP bosons should be employed to attain the excitation spectra of these phases, (see Fig. 3). In this respect, we consider a general background and divide the subsystem with spin *σ* to two sublattices with HP bosons $\hat{a}$ and $\hat{b}$, and the subsystem with spin *τ* to two sublattices with bosons $\hat{c}$ and $\hat{d}$. The vector *ψ*_k_ and the matrices *A* and *B* are given by

21 $${\psi }_{{\rm{k}}}^{\dagger }=(\begin{array}{cccccccc}{\hat{a}}_{{\rm{k}}}^{\dagger } & {\hat{b}}_{{\rm{k}}}^{\dagger } & {\hat{c}}_{{\rm{k}}}^{\dagger } & {\hat{d}}_{{\rm{k}}}^{\dagger } & {\hat{a}}_{-{\rm{k}}} & {\hat{b}}_{-{\rm{k}}} & {\hat{c}}_{-{\rm{k}}} & {\hat{d}}_{-{\rm{k}}}\end{array}),$$

22 $$A=(\begin{array}{cccc}{\alpha }_{11} & {\alpha }_{2}^{\ast } & {\alpha }_{9} & {\alpha }_{6}^{\ast }\\ {\alpha }_{2} & {\alpha }_{12} & {\alpha }_{8} & {\alpha }_{10}\\ {\alpha }_{9} & {\alpha }_{8}^{\ast } & {\alpha }_{13} & {\alpha }_{4}^{\ast }\\ {\alpha }_{6} & {\alpha }_{10} & {\alpha }_{4} & {\alpha }_{14}\end{array}),\,\,\,B=(\begin{array}{cccc}0 & {\alpha }_{1}^{\ast } & {\alpha }_{9} & {\alpha }_{5}^{\ast }\\ {\alpha }_{1}^{\ast } & 0 & {\alpha }_{7}^{\ast } & {\alpha }_{10}\\ {\alpha }_{9} & {\alpha }_{7}^{\ast } & 0 & {\alpha }_{3}^{\ast }\\ {\alpha }_{5}^{\ast } & {\alpha }_{10} & {\alpha }_{3}^{\ast } & 0\end{array}),$$

where

23 $$\begin{array}{rcl}{\alpha }_{1} & = & {w}_{ab}^{11}\,\cos ({k}_{x}a),\,\,\,{\alpha }_{2}={w}_{ab}^{12}\,\cos ({k}_{x}a),\\ {\alpha }_{3} & = & {w}_{cd}^{11}\,\cos ({k}_{x}a),\,\,\,{\alpha }_{4}={w}_{cd}^{12}\,\cos ({k}_{x}a),\\ {\alpha }_{5} & = & {w}_{ad}^{11}\,\cos ({k}_{y}a),\,\,\,{\alpha }_{6}={w}_{ad}^{12}\,\cos ({k}_{y}a),\\ {\alpha }_{7} & = & {w}_{cb}^{11}\,\cos ({k}_{y}a),\,\,\,{\alpha }_{8}={w}_{cb}^{12}\,\cos ({k}_{y}a),\\ {\alpha }_{9} & = & 2{V}_{2}\,{g}_{ac}^{1}\,\cos ({k}_{x}a)\cos ({k}_{y}a),\\ {\alpha }_{10} & = & 2{V}_{2}\,{g}_{bd}^{1}\,\cos ({k}_{x}a)\cos ({k}_{y}a),\\ {\alpha }_{11} & = & ({w}_{ab}^{23}+{w}_{ad}^{23})+2{V}_{2}\,{g}_{ac}^{2}-h{e}_{a}\mathrm{/2,}\\ {\alpha }_{12} & = & ({w}_{ab}^{34}+{w}_{cb}^{34})+2{V}_{2}\,{g}_{bd}^{2}-h{e}_{b}\mathrm{/2,}\\ {\alpha }_{13} & = & ({w}_{cb}^{23}+{w}_{cd}^{23})+2{V}_{2}\,{g}_{ac}^{3}-h{e}_{c}\mathrm{/2,}\\ {\alpha }_{14} & = & ({w}_{ad}^{34}+{w}_{cd}^{34})+2{V}_{2}\,{g}_{bd}^{3}-h{e}_{d}\mathrm{/2,}\end{array}$$

with

24 $${w}_{mn}^{\alpha \beta }={V}_{1}{g}_{mn}^{\alpha }-J{f}_{mn}^{\beta }\mathrm{.}$$

Here, *α* and *β* are 1, 2, 3, 4, and *m* and *n* are the sublattices’ labels: *a*, *b*, *c* and *d*. The coefficients ${f}_{mn}^{\beta }$, ${g}_{mn}^{\alpha }$ and *e*_*m*_ are given in terms of *θ*_*m*_*, θ*_*n*_ and *ϕ*_*m*_*, ϕ*_*n*_ as follows:

25 $$\begin{array}{rcl}{f}_{mn}^{1} & = & \sqrt{{S}_{m}{S}_{n}}((\cos \,{\theta }_{m}\,\cos \,{\theta }_{n}-\mathrm{1)}\,\cos ({{\varphi }}_{m}-{{\varphi }}_{n})+i\,\sin ({{\varphi }}_{m}-{{\varphi }}_{n})(cos{\theta }_{n}-cos{\theta }_{m})),\\ {f}_{mn}^{2} & = & \sqrt{{S}_{m}{S}_{n}}((\cos \,{\theta }_{m}\,\cos \,{\theta }_{n}+\mathrm{1)}\,\cos ({{\varphi }}_{m}-{{\varphi }}_{n})+i\,\sin ({{\varphi }}_{m}-{{\varphi }}_{n})(cos{\theta }_{n}+\,\cos \,{\theta }_{m})),\\ {f}_{mn}^{3} & = & -\,2{S}_{n}\,\sin \,{\theta }_{m}\,\sin \,{\theta }_{n}\,\cos ({{\varphi }}_{m}-{{\varphi }}_{n}),\\ {f}_{mn}^{4} & = & -\,2{S}_{m}\,\sin \,{\theta }_{m}\,\sin \,{\theta }_{n}\,\cos ({{\varphi }}_{m}-{{\varphi }}_{n}),\\ {g}_{mn}^{1} & = & \frac{1}{2}\sqrt{{S}_{m}{S}_{n}}\,\sin \,{\theta }_{m}\,\sin \,{\theta }_{n},\\ {g}_{mn}^{2} & = & -\,{S}_{n}\,\cos \,{\theta }_{m}\,\cos \,{\theta }_{n},\\ {g}_{mn}^{3} & = & -\,{S}_{m}\,\cos \,{\theta }_{m}\,\cos \,{\theta }_{n},\\ {e}_{m} & = & -\cos \,{\theta }_{m},\end{array}$$

where *S*_*m*_ and *S*_*n*_ are the spins of sublattices *m* and *n*, respectively.

In the MI(4/6), Full and SF phases where the translational symmetry of the original Hamiltonian is preserved, the classical background has a two-sublattice structure and the excitations of these phases are achieved by defining the two HP bosons: $\hat{a}$ and $\hat{d}$ for the sublattices with the spin *σ* and *τ* respectively. The Fourier transformation of the bilinear term of the LSW Hamiltonian is written by:

26 $${\psi }_{{\bf{k}}}^{\dagger }=(\begin{array}{cccc}{\hat{a}}_{{\bf{k}}}^{\dagger } & {\hat{d}}_{{\bf{k}}}^{\dagger } & {\hat{a}}_{-{\bf{k}}} & {\hat{d}}_{-{\bf{k}}}\end{array}),$$

and *A* and *B* matrices are given by

27 $$A=(\begin{array}{cc}{\alpha }_{5} & {\alpha }_{4}^{\ast }\\ {\alpha }_{4} & {\alpha }_{6}\end{array}),\,\,\,B=(\begin{array}{cc}{\alpha }_{1}^{\ast } & {\alpha }_{3}^{\ast }\\ {\alpha }_{3}^{\ast } & {\alpha }_{2}^{\ast }\end{array}),$$

where

28 $$\begin{array}{rcl}{\alpha }_{1} & = & {w}_{aa}^{11}\,\cos ({k}_{x}a),\\ {\alpha }_{2} & = & {w}_{dd}^{11}\,\cos ({k}_{x}a),\\ {\alpha }_{3} & = & {w}_{ad}^{11}\,\cos ({k}_{y}a)+2{V}_{2}\,{g}_{ad}^{1}\,\cos ({k}_{x}a)\cos ({k}_{y}a),\\ {\alpha }_{4} & = & {w}_{ad}^{12}\,\cos ({k}_{y}a)+2{V}_{2}\,{g}_{ad}^{1}\,\cos ({k}_{x}a)\cos ({k}_{y}a),\\ {\alpha }_{5} & = & {w}_{aa}^{23}+{w}_{ad}^{23}+{w}_{aa}^{12}\,\cos ({k}_{x}a)+2{V}_{2}\,{g}_{ad}^{2}-h{e}_{a}\mathrm{/2,}\\ {\alpha }_{6} & = & {w}_{dd}^{23}+{w}_{ad}^{34}+{w}_{dd}^{12}\,\cos ({k}_{x}a)+2{V}_{2}\,{g}_{ad}^{3}-h{e}_{d}\mathrm{/2.}\end{array}$$

## Summary and Conclusion

To summarize, in the present paper, employing three analytical and numerical approaches, MF approximation, CMFT with different cluster sizes and LSWT, we have studied the ground state phases of a 2D mixed-spin system of coupled alternating spin chains described by the spin Hamiltonian in Eq. (1). Our study, indicates that the CAS system displays a rich ground state phase diagram including STS and SCF phases in addition to the different solids, SF and MI phases. We have also considered two kinds of anisotropic CAS model, (*i*) CAS model with different intra-chains and inter-chains NN interactions and (*ii*) CAS model with different intra-chains and inter-chains hoppings, and investigated the effects of these anisotropies on the ground state phases. We have demonstrated that the emergence of the STS phase strongly depends on the strength of intra-chains NN interactions and hopping energies. We have shown that, for the systems with the inter-chains interactions, the repulsive intra-chains hoppings are necessary and sufficient for stripe supersolidity. In this case the minimal two dimensional mixed-spin model is a system of spin-1 and spin-1/2 XXZ chains, interacting via an Ising Hamiltonian. However, in the presence of inter-chains hoppings, the STS phase emerges even in the absence of intra-chains interactions, and a system of coupled Ising chains is the minimal model.

Our mixed-spin model is equivalent to a bosonic system of hard-core and semi-hard-core bosons and could be realized in coupled one dimensional optical lattices by alternatively changing the optical depth. Study of temperature phase diagram as well as thermodynamic properties of the CAS system and also study of the ground state phase diagram with other approaches are left for future work.
